# The Use of Ultrasound Imaging in Continuous Blood Vessel Area and Velocity Data Acquisition for Determining the Local Pulse Wave Velocity

**DOI:** 10.3390/jcm14217550

**Published:** 2025-10-24

**Authors:** Victoria Charlotte Wei Yi Ng, Hwa Liang Leo, Yoke-Rung Wong

**Affiliations:** 1 Department of Biomedical Engineering, National University of Singapore, 4 Engineering Drive 3, #04-08, Singapore 117583, Singapore; victoriacharlotte.ng@u.nus.edu; 2 Biofluid Mechanics Research Laboratory, National University of Singapore, 15 Kent Ridge Cres, #06-02 NUS Block E7, Singapore 119276, Singapore; 3 Biomechanics Laboratory, Singapore General Hospital, 20 College Road, Academia, Level 1, 20 College Road, Singapore 169856, Singapore

**Keywords:** ultrasound imaging, doppler measurement, continuous blood vessel measurements, pulse wave velocity

## Abstract

Pulse wave velocity (PWV) is a useful biomarker in the monitoring and risk stratification of various cardiovascular diseases including hypertension. The current gold standard for non-invasive measurement is carotid-femoral PWV (cfPWV) measurement via direct tonometry. However, cfPWV provides only a global PWV measure, which emphasises the need for an alternative capable of local PWV assessment. There are several alternatives for local PWV measurement proposed in the literature and one promising alternative is ultrasound, which offers good penetration depth, accessibility, and a relatively low cost, making it well-suited for non-invasive, real-time acquisition of haemodynamic parameters for PWV estimation. This paper aims to evaluate the different approaches for ultrasound-based acquisition while considering technical and physiological constraints to optimise the accuracy, reliability, and reproducibility of the parameters collected for estimation. In particular, this paper focuses on the flow-area (QA) and lnDiameter-velocity (lnDU) methods, which require local area and velocity data for PWV estimation. Accordingly, this paper discusses the use of ultrasound imaging in vessel data acquisition, highlights various challenges and considerations to be managed during acquisition and processing, outlines the different ultrasound-based imaging modalities for acquiring area and velocity data, and compares the simultaneous and non-simultaneous acquisition of data for PWV estimation.

## 1. Introduction

The pulse wave velocity (PWV) is the rate at which pressure waves propagate through the arterial system [1]. Clinically, PWV has been established as a reliable, non-invasive measure of arterial stiffness [2] and an independent risk predictor of adverse cardiovascular events including hypertension [3,4]. Subsequent explorations into the utility of PWV have led to its study as a possible parameter in the non-invasive monitoring of key cardiovascular measurements such as arterial blood pressure (ABP) [5]. In particular, the use of PWV in ABP measurement is a possible improvement to the current non-invasive clinical method of sphygmomanometry due to allowing for more continuous monitoring of ABP and therefore facilitating investigations into the dynamic behaviour of the arterial system.

The current gold standard for non-invasive PWV measurement is carotid-femoral PWV (cfPWV) measurement via direct tonometry [6,7], which involves measuring the transit time of the arterial waveform from one point on the carotid to another point on the femoral artery [7,8]. However, the global nature of the cfPWV limits the effectiveness of the method in conducting measurements over a long arterial segment. Firstly, the distance measurement of the cfPWV does not account for the path of blood from the aortic arch to the carotid artery; hence, assumptions are made to appropriately adjust the measured time taken, which limits the accuracy of the method [8,9]. Secondly, global cfPWV measurement conceals variations in arterial stiffness and mechanical properties of vessels [7,10] that may cause differences in the local PWV. Additionally, previous studies have concluded that the global PWV is less useful in providing significant clinical information than the local PWV, taken in a specific arterial segment, in terms of assessing arterial properties [11,12,13]. Hence, the utility of the cfPWV method is limited.

Considering the value of PWV measurements in the monitoring of cardiovascular health and the limitations of the global cfPWV, further research has investigated non-invasive alternatives to local PWV determination including magnetic resonance imaging (MRI) [14,15], plethysmography [4,16], and ultrasound [16].

Of particular interest among these methods is ultrasound, which leverages the presence of frequency shifts [17] in reflected high-frequency sound waves to obtain measurements at specific locations on the arterial tree. Ultrasound-based local PWV methods are indirect and require intermediate measurands for calculation and determination of the PWV. These measurands include the diameter or lumen area of the artery and the velocity of blood flow in the measured region [5,16,18,19,20,21,22,23].

Given the importance of the PWV, several previous reviews have compared a myriad of non-invasive data acquisition approaches for PWV measurement including direct tonometry, plethysmography, MRI, and ultrasound [4,16,24]. These reviews focused primarily on compiling prominent methodological information for the major acquisition methods, whereas more specific details on ultrasound optimisation for PWV acquisition have remained less explored. Meanwhile, considering the utility of ultrasound, other reviews explored developments in ultrasound techniques such as vector flow imaging and 3D flow imaging [25], while others broadly detailed ultrasound techniques for studying vascular ageing [26], with a limited discussion on considerations for developing an optimal PWV acquisition method. To date, little has been done to comprehensively discuss the optimisation of ultrasound in data acquisition for PWV estimation, particularly with respect to the flow-area (QA) and lnDiameter-velocity (lnDU) loop-based methods.

In contrast, this review seeks to evaluate different approaches for ultrasound-based acquisition while considering technical parameters and physiological realities to optimise the accuracy, reliability, and reproducibility of the parameters collected for estimation. Consequently, with a focus on the QA and lnDU loop-based methods, this paper discusses the use of ultrasound imaging in vessel data acquisition, highlights various challenges and considerations to be managed during acquisition and processing, outlines the different ultrasound-based imaging modalities for acquiring area and velocity data, and compares the simultaneous and non-simultaneous acquisition of data for PWV estimation.

## 2. Background of Ultrasound Measurement for PWV Estimation

### 2.1. Method of Processing Acquired Data

An understanding of the data-processing method used allows for a greater understanding of what data need to be acquired to improve the acquisition in the first place.

The common data processing methods used in the literature for ultrasound-based acquisition are transit time techniques and loop-based methods. Non-invasive transit time techniques include the Doppler-based cfPWV method and pulse wave imaging (PWI). Doppler-based cfPWV measurement is functionally like direct tonometry cfPWV measurement, where data are acquired via ultrasound instead of an applanation tonometer [27]. Hence, this method is not considered, as it returns global PWV measurements rather than local measurements. Meanwhile, PWI measures and images the spatiotemporal propagation of the arterial wall distension wave to compute the local PWV [28,29]. However, PWI requires very high temporal resolution to accurately capture the small wall displacements, which may not be achievable with all ultrasound machines. For instance, investigations into the temporal resolution required for PWI have shown that ultrafast frame rates of more than 2 kHz are needed for optimal measurement [29,30]. Conversely, studies with loop-based methods were able to operate with sufficient accuracy at 60–200 Hz [31,32]. Additionally, in PWI, it is assumed that the detected distension corresponds with the true forward moving wave, despite the possibility that observed distension may be due to the combination of both forward and reflection waves. Thus, this paper focuses on loop-based data processing methods which can estimate the local PWV during the reflection-free period of the cardiac cycle using current clinical ultrasound systems.

### 2.2. Loop-Based Estimation for PWV

**Figure 1:**
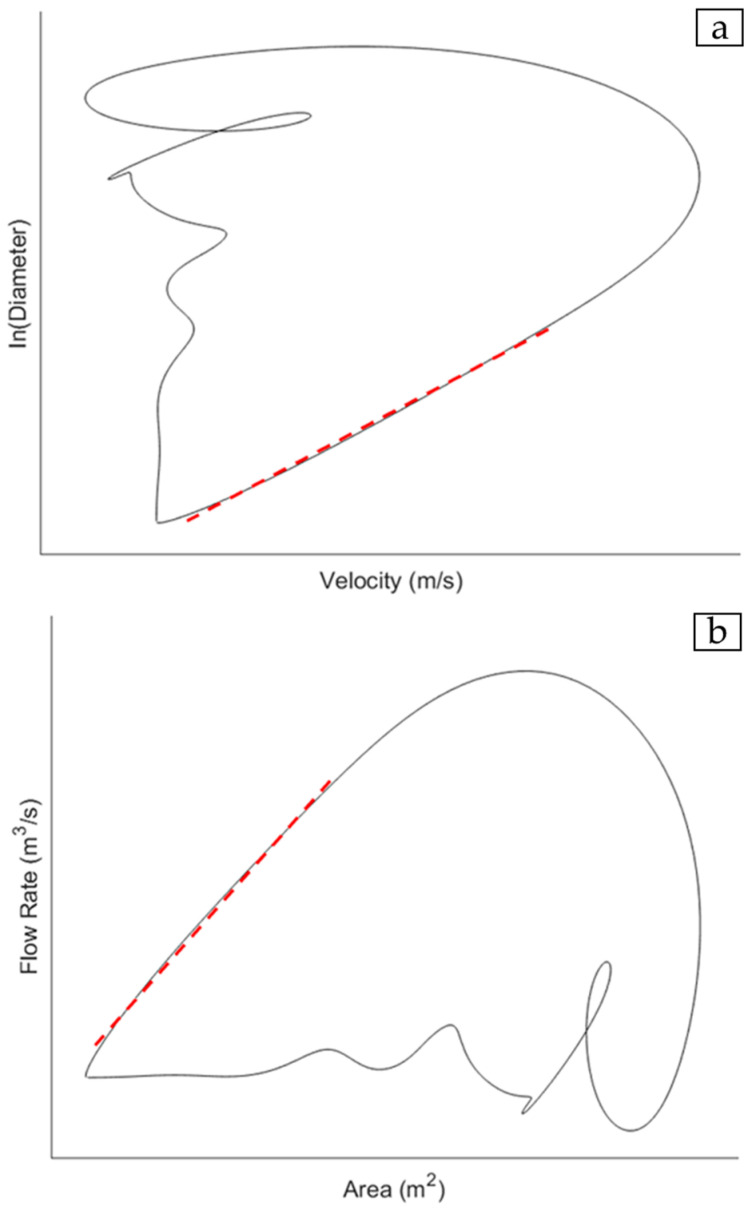
(**a**) An example of the lnDiameter-velocity (lnDU) loop depicting the linear portion of a curve (**---**) used to derive PWV via Equation (1) [21]. (**b**) An example of the flow-area (QA) loop depicting the PWV as being directly determined from the gradient of the loop at the linear portion of the loop (**---**) [19].

Loop-based methods determine the pulsatile wave from haemodynamic variables including the blood velocity and flow, the wall velocity or acceleration [5,16,18,19,20,21,22,23,28], and the vessel lumen diameter or cross-sectional area. The waveforms for a pair of haemodynamic variables are acquired over the cardiac cycle and plotted together to give a haemodynamic loop. There are two loops of interest, namely the lnDiameter-velocity (lnDU) loop (Figure 1a) [20,21,33] and the flow rate-area (QA) loop (Figure 1b) [5,19].

The full mathematical derivation of each loop can be found in [19,20] for the lnDU and QA loops, respectively. The estimation of PWV via the lnDU method is described by Equation (1):

(1) $${PWV}_{lnDU}=\pm \frac{1}{2}\frac{{dU}_{\pm }}{d{lnD}_{\pm }},$$

where *U* equals the local blood velocity within the arterial segment and *D* represents the lumen diameter of the vessel. The subscripts + and − designate the respective forward and backward directions of wave travel. In contrast, PWV estimation via the QA method is described by Equation (2):

(2) $${PWV}_{QA}=\frac{dQ}{dA},$$

where *Q* is the blood flow rate within the arterial segment and *A* corresponds to the area of the arterial lumen.

A critical assumption in both equations that affects parameter optimisation is the estimation of the PWV during the reflection-free period. For the lnDU method, earlier work describes the relationship between velocity and the natural logarithm of D as being linear and proportional to the wave speed so long as the waves are unidirectional [20,21]. Furthermore, part of the derivation of the QA equation defines the relationship between dynamic pressure and flow in the arterial system via the use of characteristic impedance, which only holds true in the absence of reflections [19]. Hence, from both the loops, the PWV is determined based on the gradient of the linear portion of the curve which corresponds to the early systolic period in the cardiac cycle, which is widely accepted as being reflection-free [34,35]. Previous studies demonstrated that both loops could detect changes in the local PWV stemming from cardiovascular diseases with sufficient accuracy [19,21,31,36]. The QA method is conceptually direct, being derived from the fundamental flow–area relationship of the water-hammer equation that links local pressure, flow, and area changes [19]. Conversely, the lnDU method is computationally simpler, with higher reproducibility and operational consistency in practice [21,31]. Since both are widely used in local, loop-based PWV estimations and rely on similar measurands, both are considered in this paper within the context of acquisition optimisation.

Another loop method involves the acquisition of pressure as a parameter and includes the pressure–velocity and pressure–flow rate methods [37]. However, unlike area and velocity, current ultrasound machines do not have a means to measure pressure directly; hence, the pressure loop methods are not considered in this paper.

### 2.3. Functioning Principles Behind Ultrasound Measurement

Ultrasound data acquisition involves the use of high-frequency longitudinal pressure waves or ultrasound pulses, typically in the diagnostic range of 2–15 MHz [38], for measurement. During acquisition, the ultrasound pulses are generated and detected by a transducer. During transmission, a fast-alternating electric field is applied to the piezoelectric crystal within the transducer head, causing the crystals to vibrate at ultrasound frequencies and emit pulses into the body. During detection, the process is reversed. At the interface between media of differing acoustic impedances, the ultrasound pulse is reflected toward the piezoelectric crystal, vibrating it. Subsequently, the vibration from the returned echo is converted into electrical radiofrequency (RF) signals. By analysing the echo arrival times from successive tissue boundaries, changes in arterial lumen area and diameter can be measured for PWV estimation [39]. For such imaging, the size of the object should be larger than the pulse wavelength to produce clear reflected echoes. In contrast, objects smaller than the pulse wavelength, such as erythrocytes, generate weak, diffuse Rayleigh scattering that results in indistinct images. However, the same scattering provides the basis for velocity and acceleration measurements taken via Doppler ultrasound [40]. In Doppler ultrasound, a small component of the scattered signal from each moving erythrocyte is detected by the transducer. This combined backscattered signal produces a measurable echo with a frequency shift that is used to calculate velocity.

Recent advances in ultrasound-based PWV estimation have arisen from improvements in both acquisition methodology and system electronics and computation. Methodological developments involve the use of simultaneous or non-simultaneous data acquisition, the number and positioning of transducers used [41], and differing data processing methods. In parallel, upgrades in system electronics and computation aim to enhance the reliability of signal acquisition by improving the efficiency, bandwidth, sensitivity, and stability of transducers [42], as well as improving their frame rates to achieve better temporal resolution [16]. These upgrades encompass enhancements to existing transducer arrays [26,43] via modifications of the materials used for the piezoelectric component [42] and the adoption of advanced scanning techniques such as plane wave insonification, coherent spatial compounding, multiline transmission, and motion matching techniques [16].

### 2.4. Comparative Advantages of Ultrasound Imaging in Non-Invasive PWV Acquisition

Among clinically available imaging techniques, ultrasound presents distinct advantages for non-invasive PWV acquisition, including good penetration depth [44], real-time imaging capability [45,46], and its established use in cardiovascular monitoring [47].

As a clinical imaging modality, ultrasound is relatively low-cost compared to other methods such as computed tomography (CT) and MRI [48]. Additionally, current ultrasound machines allow for repeated imaging of the arterial segment of interest without exposure to ionising radiation and the variety of transducers available enables imaging and data acquisition from both superficial arteries like the common carotid arteries [49,50] and deeper central arteries like the abdominal aorta [51].

There are also several factors that make ultrasound more favourable than MRI, which is considered another non-invasive alternative in routine PWV measurement. Apart from the cost, a limiting factor of MRI’s utility compared to ultrasound is the long duration [52,53,54] and the need for breath-hold in traditional MRI acquisition to minimise noise during acquisition [15]. Breath-hold usually occurs over a period of 10–20 s and may not be possible in some patients including individuals with shortness of breath or cardiac failure [15]. Recent developments in MRI technology such as respiratory gating and motion-correction algorithms have enabled free-breathing during acquisition [55]. However, these techniques do not eliminate motion blurring [56,57] and usually sacrifice some spatial and temporal resolution for image stability [58], which is not ideal for precise waveform acquisition in PWV estimation. Moreover, these techniques require advanced reconstruction in imaging, which increases the computational time and burden [59,60]. Hence, despite advancements in free-breathing MRI, ultrasound remains more practical for real-time, routine clinical acquisitions.

In terms of the quality of the acquired data, ultrasound has better temporal and spatial [61] resolution than MRI and is effective in capturing real-time functional information [62]. This makes ultrasound more suited to capturing the minute changes in distension of the arterial lumen and changes in blood velocity over each cardiac cycle for PWV estimation.

Moreover, in comparison to the large, generally immovable machinery used in MRI, ultrasound machines are more compact and portable [63], with ultrasound being used regularly in hospitals and clinics for cardiology and obstetrics [64]. Furthermore, improvements in ultrasound transducer technology have also led to the creation of handheld transducers that can be connected directly to smartphones [65], thereby further increasing the accessibility of ultrasound.

These advantages make ultrasound favourable for clinical applications in the routine measurement of PWV and monitoring of cardiovascular health.

## 3. Key Challenges and Considerations in Ultrasound Imaging for PWV Measurement

In acquiring parameter data for PWV estimation, two categories of issues arise. These include challenges, which are intrinsic sources of variability in measurement that must be mitigated, and considerations, which are adjustable software or hardware factors that can be controlled to optimise data acquisition. In this section, possible mitigation strategies for the identified challenges are proposed to improve acquisition and processing to obtain greater PWV estimation accuracy.

### 3.1. Physiological Challenge: Wave Reflections

As previously mentioned, a key challenge in the use of the haemodynamic loops for processing the acquired data is the presence of reflected waves. Wave reflections occur due to impedance mismatches in the arterial tree such as at sites of bifurcation and tapering, where the vessel size decreases in the periphery [66,67]. The presence of backward reflected waves complicates PWV calculations that assume forward blood flow, thereby causing an undesired divergence in the pressure and flow waveforms [35,67] and affecting PWV estimations [68]. As discussed, current loop methods attempt to mitigate this issue through estimation at the early systolic reflection-free period of the cardiac cycle [5]. Yet, the presence and duration of the reflection-free period vary depending on the imaged vessel [69,70]. This limits the feasibility of the ultrasound-based data acquisition to central arteries, as peripheral vessels have significantly shorter reflection-free periods, although it is argued that the use of high sampling rates and knowledge of wave speed can assist with limiting analysis purely to forward flow [5].

Alternatively, wave separation analysis (WSA) techniques may be employed post-acquisition to separate forward and backward flow. Manoj et al. [71] demonstrated that adopting WSA techniques reduced the variability in PWV estimation by 65% in ultrasound-based acquisition and yielded values that aligned closely with theoretical PWV values. Thus far, WSA has been applied to transit time techniques rather than loop-based methods for PWV estimation, yet it is reasonable to propose the extension of WSA to loop-based approaches by applying separation prior to constructing loops from forward-only components. This avenue remains unexplored and requires further validation.

Overall, mitigation of wave reflections is crucial in improving the accuracy of acquired data and incorporating WSA may aid in addressing this challenge.

### 3.2. Physiological Challenge: Differences in Acoustic Impedance of Internal Structures

Differences in acoustic impedances within the human body represent another significant intrinsic physiological challenge that needs to be managed. Given that reflections in ultrasound are generated at tissue boundaries where the acoustic impedances differ, significant differences in acoustic impedance such as those between tissue, bone, and gas can cause unwanted artefacts [72] like shadowing that prevent imaging of deeper tissue structures. This has implications on the feasibility of ultrasound imaging at different arteries. For instance, the thoracic aorta, which is surrounded by the rib bones and gas in the lungs, is typically not imaged by non-invasive ultrasound methods [73,74]. Meanwhile, while ultrasound imaging of the abdominal aorta is feasible, bowel gas may cause undesired artefacts that affect imaging [51,75]. Managing acoustic impedance differences within the body may involve selecting a relatively less obstructed artery such as the common carotid artery (CCA) or compensating for reflectors that block ultrasound access to the artery. For example, the effect of bowel gas on abdominal aorta imaging may be mitigated with fasting prior to imaging [76] or with probe pressure during examination to rapidly move the gas [77].

### 3.3. Technical Challenge: Operator Skill Issue

A challenge intrinsic to ultrasound measurement is operator skill, as this modality is highly operator-dependent during both image acquisition and data interpretation [78]. This operator dependence presents a risk of accuracy reduction and can impact the intra- and inter-operator repeatability of measurements [79]. Given the prevalent clinical use of ultrasound [80], several studies have examined methods to tackle reliance on operator skill.

In vessel image acquisition, automatic wall tracking methods assist in obtaining more accurate, precise, and consistent measurements of vessel diameter changes over the cardiac cycle [81,82,83,84]. In Doppler ultrasound for velocity or volumetric flow measurements, it was suggested that greater operator independence may be achieved using 3D Doppler ultrasound that captures volumetric data rather than the velocity data in a specific plane to reduce the need for precise transducer alignment [85]. However, despite these purported improvements, the method still relies on Doppler angle correction by the operator, wherein inaccuracies in angle estimation can introduce velocity measurement errors [86]. Moreover, 3D Doppler measurement reduces but does not eliminate the fact that a poorly aligned transducer relative to the vessel can cause signal degradation and measurement errors. While these methods assist in reducing issues due to operator skill, they do not fully mitigate issues that arise from poor positioning, the angling of transducers, or sub-optimal selection of the region of interest in ultrasound technology.

A future approach to tackling operator dependence is the use of machine learning to assist in data acquisition. Machine learning systems may be able to provide real-time feedback or guidance to operators during image acquisition itself along with image quality control and automatic rather than manual selection of the vessel region of interest [46]. For instance, deep learning methods have been proposed for automatic Doppler angle estimation from B-mode imaging to reduce operator bias [87].

### 3.4. Software-Based Considerations

Prior to data acquisition, it is crucial to optimise key software parameters including the frame rate, pulse repetition frequency (PRF), gain, dynamic range, and filter settings, which have been identified to more greatly affect PWV data acquisition. The adjustment of each parameter should account for acceptable compromises, as improvement in one aspect often causes deterioration in another.

#### 3.4.1. Frame Rate

The frame rate, defined as the number of image frames per second, directly affects the temporal resolution [88]. In parameter acquisition, a sufficiently high temporal resolution is essential to capturing arterial changes during the short duration of the cardiac cycle, as accurate PWV estimation relies heavily on precise timing of the parameter waveforms. The frame rates utilised in previous studies ranged from 60–200 Hz [19,31,32]. The frame rate can be increased by reducing the imaging depth, narrowing the width of the region of interest, and decreasing the line density [89].

#### 3.4.2. Pulse Repetition Frequency

Pulse repetition frequency (PRF) refers to the number of ultrasound pulses emitted over time. The PRF can be adjusted manually to minimise or eliminate aliasing, which otherwise causes inaccuracies in velocity measurement for PWV estimation. In Doppler imaging, adjustments should be made to ensure that the blood flow velocity is below the Nyquist limit, which is equal to half the PRF. During adjustments, higher PRF values reduce the effects of aliasing at the cost of shallower penetration depths [90]. In medical imaging, the typical PRF range is 1–10 kHz [91,92] and the optimal PRF value varies between patients but is often within a certain range [93]. For instance, at shallower arteries such as the CCA, previous studies have suggested the use of PRF values in the 3–4 kHz range [91,93]. Conversely, at deeper locations such as the abdominal aorta, a lower PRF in the range of 1–3 kHz is recommended [91]. The PRF can be tuned by identifying aliasing, which appears as abrupt cut-offs with inversions in the spectral trace or mixed colours in colour Doppler. Once aliasing is identified, incremental adjustments to the PRF can be made until the spectrum is continuous and the colour display is uniform.

#### 3.4.3. Gain Function and Time Gain Compensation

The gain function amplifies echoes to overcome attenuation and increase brightness. However, excessive gain increases the amount of noise and decreases the signal–noise ratio [38]. Meanwhile, time gain compensation (TGC) selectively amplifies signals at greater depths without increasing the noise in more superficial layers [38]. The fine-tuning of the gain and TGC is dependent on the vessel location and the tissue composition of the subject. The gain is usually adjusted from preset values to modify the overall brightness of the sonogram, whereas TGC controls the depth-specific brightness and can likewise be adjusted from preset values [94]. In practice, the tuning of both parameters is determined by visual confirmation of the optimal contrast within the region of interest. Taken together, effective tuning of both the gain and TGC improves the imaging contrast and ensures consistent arterial wall detection in diameter and area measurement.

#### 3.4.4. Dynamic Range

The dynamic range is the range between the maximum and minimum values of the displayed signal [95], which affects the clarity of the displayed lumen wall. A low dynamic range for arterial lumen wall measurement is generally preferred [96], as it provides a suitably high contrast between the vessel wall and the lumen. Given that the dynamic range adjusts how the signal amplitudes appear visually, fine-tuning of the dynamic range should be performed with visual confirmation of high contrast in B-mode imaging.

#### 3.4.5. Filters

Filters enhance the signal quality by suppressing unwanted components and improving the signal–noise ratio. Among the filters available on clinical ultrasound systems, three have the most pronounced influence on the optimisation of parameter acquisition. The wall filter removes low-frequency motion components to isolate blood flow in Doppler imaging [97], while the persistence filter averages consecutive frames to stabilise noisy images at the possible expense of temporal resolution [98]. The speckle-reduction filter improves the visual clarity of lumen boundaries but, if applied in excess, can decrease the resolution of images of the arterial wall [99]. Hence, while useful in improving acquisition accuracy, these filters must be carefully optimised to avoid the unintended erasure of relevant physiological flow components.

### 3.5. Hardware-Based Considerations

#### 3.5.1. Coupling Media

An important hardware-based consideration for ultrasound imaging is the use of coupling media during imaging. For the signals to be optimally transmitted to the organ of interest, the transducer head must be in full contact with the skin surface, as the presence of air pockets between the transducer head and the skin surface will cause large acoustic impedance differences and prevent ultrasound transmission for imaging. Hence, a coupling medium is employed to decrease the difference in acoustic impedance [100]. The gold standard for media in clinical imaging is ultrasound gel, which is spread over the measurement location. Although the gel is a fast and convenient option, it may spread inconsistently, particularly for irregular surface anatomy, and reapplication might be needed for wider area measurement. For acquisitions on irregular surfaces such as the neck, a gel pad is a possible alternative. During acquisition, the pad is laid over the area of interest or attached to the transducer head to provide a consistent and wide region for scanning [101]. An alternative to gel media is water baths, in which the whole body or body part is submerged into water [102,103]. The water bath method has been shown to provide superior imaging quality when compared to gel-based imaging, particularly in peripheral vessels, and removes the need for direct skin contact [63]. However, water immersion for imaging may be less feasible for imaging certain arteries such as the CCA, whose measurement location is at the neck.

#### 3.5.2. Transducer Choice

Another hardware-based consideration is the transducer choice. A wide array of transducers is available for data acquisition and the decision of which type of transducer to use depends largely on the desired measurement site. More superficial measurement locations have a lower depth of focus and can be imaged with higher-frequency transducers that offer better resolution with a smaller penetration depth. Conversely, deeper measurement locations have a greater depth of focus and demand lower-frequency transducers that afford greater penetration depths with poorer resolution. For instance, PWV measurement in the abdominal aorta may require a 3.5 MHz transducer that provides sufficient tissue penetration (>15 cm) for imaging at the cost of lower image resolution [38]. In contrast, it is more appropriate to use a higher 7.5 MHz frequency transducer [31] that provides a lower penetration depth but higher image resolution for measurement in the more superficial CCA.

Having outlined the key physiological and technical challenges and considerations that influence the acquisition of arterial parameters for PWV estimation in practice, the following sections examine the ultrasound modes for area and velocity measurement and evaluate the simultaneous and non-simultaneous uses of these modes that operate within the discussed constraints.

## 4. Ultrasound Imaging Modes for Vessel Area Acquisition

Vessel area acquisition using ultrasound typically involves the transverse placement of the transducer to obtain a cross-sectional view of the arterial lumen. However, while transverse placement is useful for anatomical localisation, it is unsuitable for use in PWV estimation, particularly with loop-based methods, due to the plane mismatch in parameter acquisition. Doppler measurements for velocity and flow, as discussed in later sections, require the transducer to be positioned longitudinally for alignment with the direction of blood flow. Given that the area waveform should be acquired within the same segment of the vessel and appear in the same sagittal plane for the subsequent alignment with the velocity or flow waveform to be optimal, a longitudinal placement along the vessel axis is preferred [5,33,104].

As such, since longitudinal measurements provide a one-dimensional view of the vessel, the vessel distension over the cardiac cycle is measured via changes in the diameter of the vessel lumen. The geometry of the vessel lumen is assumed to be axisymmetric and circular, with loop-based PWV estimation studies typically modelling the vessel segment as a uniform elastic cylinder [19,32,105,106]. Subsequently, loop methods requiring cross-sectional area measurements derive the area from the diameter via the equation, $A={\pi (D/2)}^{2}$.

Therefore, the following section explores area measurement techniques that involve longitudinal transducer placement and the technical developments that improve the accuracy, reliability, and repeatability of acquisition.

### 4.1. Imaging Modalities

**Figure 2:**
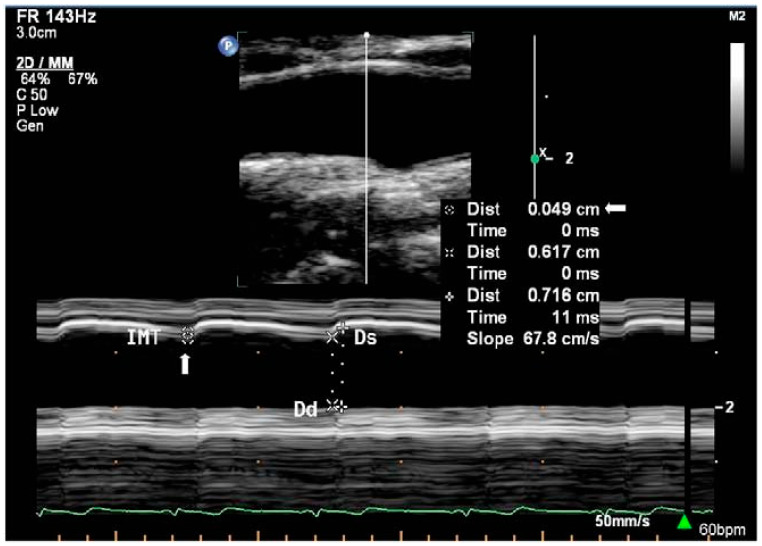
M-mode imaging of CCA with graphical trace of diameter changes below the B-mode image [110]. Reprinted from Perspectives in Medicine, Volume 1, Issues 1–12, Galinda Baltgaile, Arterial wall dynamics, Pages 146–151, Copyright (2012), with permission from Elsevier.

In area or diameter measurement, sound waves emitted by the transducer are partially reflected and transmitted at tissue boundaries with different acoustic impedances in the beam path, which causes differences in the echo return time and facilitates detection of different tissues at different depths [38]. There are three main imaging modes available on ultrasound machines, namely Amplitude (A-mode), Brightness (B-mode), and Motion (M-mode). A-mode ultrasound provides a one-dimensional scan line that displays the strength of the echoes as vertical peaks on a graph. While some studies have explored the use of A-mode ultrasound in simple system configurations to measure diameter [107,108,109], its use remains relatively under-represented in the literature relating to diameter and area acquisition. Hence, only B-mode and M-mode (Figure 2) ultrasound will be discussed. The functioning principles and use of each modality in measurement are summarised in Table 1.

### 4.2. Developments in B- and M-Mode Imaging

Developments in B- and M-mode imaging for area and diameter acquisition revolve around improving the resolution and contrast and involve technical improvements peri- and post-acquisition. This section briefly discusses the various explored categories of improvement to B-mode and M-mode imaging during and after acquisition which aid in improving the accuracy, reliability, and repeatability of acquisition for PWV estimation.

Peri-acquisition developments are techniques applied during acquisition to influence real-time image formation. These improvements are focused on increasing frame rates and contrast through techniques such as ultrafast plane-wave imaging [111,112] and spatial and frequency compounding [113,114], while reducing the effects of speckle artefacts via adaptive and coherence-based beamforming [111,115].

At the post-acquisition stage, advancements in digital signal and image processing refine raw RF data to improve image quality. For B-mode imaging, deconvolution techniques such as higher-order statistics [116] and autoregressive parameter modelling [117] have improved the spatial resolution and contrast ratio of images. Concurrently, various non-linear beamforming techniques such as Filtered Multiply and Sum (FMAS) [118], Delay Multiply and Sum (DMAS) [119], and Cross-Angular Delay Multiply and Sum (CADMAS) [120] have been devised to enhance image contrast. In M-mode imaging, technical developments such as temporal smoothing and interpolation focus on optimising motion analysis.

Taken together, these peri- and post-acquisition developments enable greater accuracy and reliability in the tracking of arterial wall distension. Therefore, consideration of the use of one or a combination of the available techniques to optimise diameter and area acquisition is warranted.

### 4.3. Automatic Wall Distension Tracking Methods

In the measurement of vessel diameter, inbuilt digital callipers can be used to manually mark the desired region of interest for measurement. However, such manual tracking of wall distension in either B-mode or M-mode can be problematic due to its time-consuming and subjective manner, where small deviations in placement at the measured region of interest, due to visual identification, can cause large errors in area measurement. Hence, developments of more objective and automatic wall tracking methods are crucial for PWV estimation.

Owing to its necessity, wall tracking for ultrasound-based methods has been studied in detail, from the use of threshold detectors to study echoes from the arterial wall [121], which is limited due to dependence on the distance to the arterial wall, which varies through the arterial cycle, to methods that use phase-locking devices which track a specific vessel wall echo [122,123]. Further developments also investigated combining the tracking system with B-mode imaging [124] and pressure recordings [125]. Alternatives to these phase-locking methods were also studied, alongside the use of conventional autocorrelation, which is independent of radiofrequency (RF) centre frequency [126,127] but requires lower sampling rates, and subsequently RF cross-correlation [128,129]. Cross-correlation techniques, however, have an estimator bias that depends nonlinearly on the actual displacement [81]. To address this, a modification was made to the autocorrelation method to estimate the mean Doppler frequency and RF centre frequency for wall tracking [130,131]. In this modified method, wall tracking is conducted by integrating wall velocities as estimated by Doppler techniques. This method was found to track wall vessel motion with lower bias and variance than previous cross-correlation methods, and thus improves tissue tracking accuracy [81].

Another type of algorithm developed to measure vessel diameter using B-mode imaging utilises the differences in pixel intensity values or brightness within a user-defined region of interest to determine the wall location and hence measure the diameter [82,84]. To accurately determine the wall location, the transducer is placed longitudinally to the vessel. During acquisition, the resolution of the images must be sufficiently high to observe the vessel wall layers. Additionally, electrocardiogram (ECG) gating may be employed to capture images over the cardiac cycle, with the R signal being used as a reference point. An advantage of this algorithm is its purported simplicity that facilitates possible integration into current clinical machines while being robust to differences in image quality [82].

In automatic wall tracking, algorithms also exist for diameter measurement using a transducer placed transverse to the vessel [83]. However, as mentioned, transverse transducer placement is not optimal for subsequent velocity measurement and movement of the transducer may cause changes in the measured region; hence, such wall tracking methods are less useful in PWV acquisition.

## 5. Doppler Ultrasound Imaging Modes for Velocity Acquisition

Acquisition of velocity for PWV estimation is conducted using Doppler ultrasound. Akin to area acquisition in the previous section, the volumetric blood flow in loop-based PWV estimation, specifically the QA method, is not measured directly via ultrasound imaging but derived from velocity measurements [5]. Hence, this section explores the technical developments in the various Doppler imaging modalities as well as the higher-level approaches to measuring velocity that utilise Doppler ultrasound.

### 5.1. Doppler Imaging Modalities

**Figure 3:**
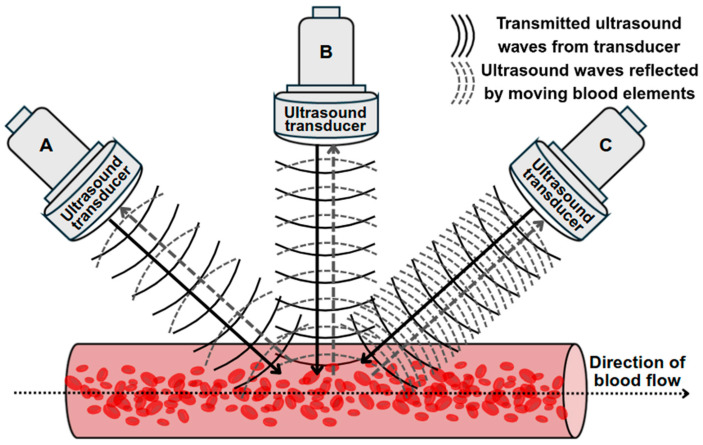
Doppler shifts with transducers at different angles. Frequency increases when blood flows toward transducer (C) and decreases with flow away from the transducer (A). There is no change in frequency when the transducer is perpendicular (B) to the vessel.

In velocity or acceleration measurement, the Doppler ultrasound mode leverages the change the frequency of the sound wave, or Doppler shift, due to reflectors such as blood elements moving towards or away from the transducer, to perform measurement (Figure 3) [132]. This frequency shift is proportional to the velocity [132]. In Doppler imaging, the magnitude and presence of the Doppler shift are affected by the Doppler angle, which is the angle between the ultrasound beam and the direction of blood flow [132]. As mentioned, the Nyquist limit is crucial in imaging for the accurate measurement of velocity via Doppler ultrasound. Beyond the Nyquist limit, undesirable aliasing occurs which can appear as incomplete peaks in the velocity curve trace for spectral Doppler imaging or as a mixture of colours in the imaged vessel under colour Doppler mode [133].

There are four main Doppler modes that can conduct velocity measurement, including spectral Doppler, colour Doppler, pulsed wave (PW) Doppler, and continuous wave (CW) Doppler, and these are summarised in Table 1. In the context of PWV estimation, CW Doppler is not a preferred mode to acquire velocity as it provides measurements of velocity along the entire beam path without the depth localisation needed for quantitative velocity measurement.

### 5.2. Developments in Doppler Imaging Modes

For current Doppler modalities, technical developments are focused on post-processing techniques to refine raw Doppler signals to obtain improved velocity measurement. This section briefly discusses the developments in post-processing that improve the accuracy of velocity measurements.

In Doppler measurement, angle and calibration corrections are conducted to reduce the cosine-angle error and improve the velocity measurement accuracy. The cosine-angle error stems from the reliance of the Doppler effect on the cosine of the angle between the ultrasound beam and flow direction. When the angle is not parallel to the flow direction, the measured signal is reduced. While commercial ultrasound systems have in-built angle correction features to compensate, these features provide insufficient correction in complex vessel geometries during velocity acquisition [134], which thereby justifies the need for more advanced corrections, especially in PWV estimation where accurate velocity is vital. Hence, more complex angle correction and calibration methods such as dual-beam ultrasound, which removes the influence of the Doppler angle on the velocity measurement [135], are needed. Other technical developments applicable across the Doppler modes include the use of the previously discussed filters to improve the signal–noise ratio and increase the accuracy of the measured velocity [136].

Beyond angle corrections and filtering, the recent literature has increasingly leveraged machine learning and deep learning to improve the accuracy of measurement by focusing on Doppler denoising, aliasing correction, and velocity and angle estimation. For instance, deep neural networks have been used for contrast enhancement [137], denoising, and aliasing correction [138,139], while different convolutional neural networks (CNNs) have been applied to facilitate high-frame-rate colour Doppler acquisition under constrained sampling to achieve improved velocity estimation [140]. Concurrently, another approach leveraged a combination of blind deconvolution and robust principal component analysis to improve Doppler measurements by separating signals from clutter and blood flow components [141]. Collectively, these machine learning developments enable more accurate velocity and flow measurements, which is crucial for local PWV estimation.

### 5.3. Doppler-Related Approaches to Velocity Acquisition

Advancements in ultrasound-based velocity acquisition have also emerged via refinement of more complex Doppler ultrasound techniques used to acquire velocity.

One such technique is vector flow imaging (VFI), which quantitatively evaluates the direction and magnitude of the true velocity vector and is an angle-independent estimation of flow velocity [142]. VFI has been shown to consistently track omnidirectional pulsatile flow [143] with enhanced sensitivity in evaluating structural and functional changes within major arteries such as the carotid [144] and has enabled greater accuracy in tracking the flow profile within tortuous vessels [25]. In the context of velocity acquisition for PWV estimation, VFI facilitates the real-time visualisation of complex flow patterns with very high frame rates [25], thereby enabling the acquisition of velocity data at higher temporal resolutions. The angle-independent nature of VFI also reduces operator bias during velocity acquisition. While VFI application in velocity acquisition requires a high computational demand, it might still be feasible for use within a limited-size region of interest like a specific arterial segment for local PWV estimation [145]. However, exploration into the efficacy of VFI in loop-based PWV estimation is relatively limited.

Contrast-enhanced Doppler is another technique developed to improve accuracy of Doppler measurements. This technique requires the injection of microbubbles as contrast agents that amplify the backscattered signals, which increases sensitivity [146]. In PWV estimation, the increased sensitivity facilitates acquisition of signals for velocity measurement in deeper arteries such as the abdominal aorta and in arteries with lower velocity flow [147].

Having summarised the acquisition modalities and their technical developments that enable greater accuracy, reliability, and repeatability of area and velocity acquisition, the subsequent section details, compares, and evaluates the simultaneous and non-simultaneous methods by which these developments can be incorporated in the context of PWV estimation.

**Table 1:** Summary of ultrasound imaging modes for data acquisition.

ULTRASOUND IMAGING MODE FOR VESSEL AREA ACQUISITION			
**IMAGING** **MODALITY**	FUNCTIONING PRINCIPLE	USE OF MODALITY	REF.
B-mode (Brightness mode)	Cross-sectional (two dimensional) image of body made of multiple scanlines. Each scanline is made up of multiple points and corresponds to the relative position where transmitted echo returned from. Image brightness is relative to echo strength returning from each point.	The vessel area is determined from the measured diameter. Diameter is measured in the longitudinal plane as distance between the anterior and posterior walls in B-mode. Use digital callipers for manual diameter measurement or wall tracking algorithms for automated measurement	[82,148]
M-mode (Motion mode)	One dimensional display of motion over time. Relates ultrasound wave amplitude to imaging of moving structures. Used for fine measurements.	Focus in one dimension in M-mode allows for higher temporal and spatial resolution. M-mode imaging displays the displacement of a detected tissue boundary graphically (Figure 3) for diameter measurement	[149]
**DOPPLER ULTRASOUND IMAGING MODES FOR VELOCITY ACQUISITION**			
**IMAGING** **MODALITY**	**FUNCTIONING PRINCIPLE**	**USE OF MODALITY**	**REF.**
Spectral Doppler	Doppler effect is used to analyse spectrum of frequencies in ultrasound echoes	Used in vascular imaging Graphical representations of velocity over time Determine velocity at different timepoints	[150]
Colour Doppler	Measurements of velocity and direction of blood flow are used to superimpose a colour pattern onto the B-mode image	Two complementary colours represent flow toward and away from the transducer Colour shades represent higher (lighter) or lower (darker) velocities An intermediate colour (green) indicates flow turbulence.	[149]
Pulsed wave (PW) Doppler	Allows for the measurement of velocity at a specific location at a specific tissue depth Allows for precise blood flow measurement.	Requires high PRF Frequency of the transducer used should be compatible with the depth of tissue measured (i.e., higher frequencies for superficial structures) Transducer angle should be less than 60°	[149,150]
Continuous wave (CW) Doppler	Uses separate piezoelectric crystal elements to transmit and receive ultrasound	Sensitive measures velocity along entire ultrasound beam Does not return specific information on depth, direction of flow or velocity.	[150]

## 6. Non-Simultaneous Versus Simultaneous Area and Velocity Acquisition

This section compares and evaluates ultrasound-based data acquisition methods for PWV calculations made via haemodynamic loop approaches. As previously discussed, the loop methods, namely QA and lnDU, both require the acquisition of velocity and diameter from the artery of interest. However, there are differences in how data are acquired, whether simultaneously or non-simultaneously. Unlike the preceding sections, which focused on optimising the acquisition of each parameter individually, this section describes and evaluates acquisition methodologies for loop-based PWV estimation.

### 6.1. Non-Simultaneous Acquisition Methods

Previous studies employing non-simultaneous acquisition have generally relied on repositioning a single transducer to obtain separate area and velocity recordings [19,31,151]. The transducer is adjusted perpendicularly for area measurements and subsequently tilted to acquire Doppler velocity data while appropriate angle corrections are applied.

An early non-simultaneous acquisition method utilised in [31] employs B-mode imaging for diameter acquisition and PW Doppler imaging for velocity acquisition before using the lnDU loop for PWV estimation. This method involves obtaining B-mode images with the region of interest in the focal zone, where images should clearly capture anterior and posterior walls, to reduce noise and obtain optimal diameter curve measurement. Subsequently, using the same scan projection, PW Doppler measurement is conducted with as small an angle correction as possible to prevent shifts in the scan location [31]. In this method, angle correction is needed, as Doppler imaging requires the Doppler angle for frequency shifts and velocity measurement. Studies employing this methodology typically use high-frame-rate electrocardiogram (ECG) gating to prevent a mismatch in features of both diameter and velocity curves over each cardiac cycle by allowing for time alignment, which uses the ECG to set a trigger signal [151,152].

In contrast, Rabben et al. [19] proposed a different non-simultaneous acquisition method that utilises M-mode imaging instead of B-mode imaging to record ultrasound data over three to five cardiac cycles for diameter acquisition. Akin to the previous method, after diameter acquisition, PW Doppler imaging is used to extract maximum velocities across the vessel before using the QA loop for PWV measurement. To ensure proper alignment, the intima layers for both anterior and posterior walls should be clearly visible. According to [19], in this non-simultaneous acquisition method, the adjustment of the transducer allowed for optimal data collection, wherein the diameter distension measurements were of high accuracy and precision.

Together, these findings highlight that, while non-simultaneous setups are practical, their susceptibility to alignment errors motivates exploration of simultaneous methods.

### 6.2. Simultaneous Acquisition Methods

Simultaneous acquisition of diameter and velocity data is technically more complex than non-simultaneous acquisition owing to the need for acquisition of both the velocity and diameter at the same transducer angle. This has been explored in studies seeking to overcome alignment errors.

For instance, a method utilised in [33,104,153] employed M-mode for diameter measurement and PW Doppler for velocity measurement. In these studies, prior to data acquisition, the transducer was positioned longitudinally in B-mode while ensuring that the vessel walls were clearly delineated. The Doppler angle used was near optimal at 58 to 60 degrees and the Doppler gating was adjusted in B-mode as well. Subsequently, during measurement, a split screen was used to obtain both diameter and velocity data at a sampling frequency of 1000 Hz. Although this method allows for automatic acquisition of the data simultaneously via the split screens, the gates and angles still need to be adjusted manually, which may affect the signals, particularly in the derivative calculations used to obtain the PWV from the lnDU loop [33,104]. This method was also used in separate research that tested a position near the aortic arch, wherein 10-beat cine loops were used in M-mode and PW Doppler imaging. Kowalski et al. [153] further introduced the use of a fluid-filled phantom between a probe and vessel which acted as an intermediate medium to prevent external pressure from the transducer from distorting the aortic wall motion.

Simultaneous acquisition, as proposed in [5], may also be conducted with constant perpendicular placement of the ultrasound transducer via the perpendicular ultrasound velocimetry (PUV) technique. In this technique, the velocity is not obtained via Doppler ultrasound but rather via cross-correlation techniques. The principle for measurement is akin to particle imaging velocimetry (PIV) [154] and PUV uses 2D cross-correlation in the time domain on raw RF data to determine the axial velocity distribution of the flow. In PUV, the ultrasound system is operated in fast B-mode or multiple M-line mode. According to Beulen et al. [5], the perpendicular placement allows for accurate and simultaneous assessment of diameter and velocity without the need to account for angle correction.

Rowland et al. [34] introduced a method similar to PUV which obtains measurements of diameter and velocity from B-mode images. In this study, one-dimensional (1D) cross-correlation techniques, rather than 2D cross-correlation, were employed to track the wall motion and blood element speckle motion in the successive B-mode images [34]. For diameter measurement, 1D cross-correlation of successive frames was conducted separately for anterior and posterior walls and the wall motion waveform for each wall was given by the cumulative summation of displacements over time. Thereafter, the diameter change waveform was obtained by the difference in wall displacement for anterior and posterior wall motion waveforms. The absolute diameter waveform was subsequently obtained with knowledge of the initial vessel diameter without distension. Meanwhile, the velocity was determined by tracking and cross-correlating the movement of the blood speckle pattern between frames, with the highest correlation indicating the mostly likely location of blood element movement [34]. In this method, knowledge of the displacement and imaging frame rate allows for the velocity to be obtained. For accurate measurement, an ultrafast scanner was used to adequately resolve the rapid acceleration and deceleration of blood during systole [34]. Akin to previous methods, ECG gating was used for time alignment while singular value decomposition (SVD) was applied to separate weak blood and strong tissue signals [34].

**Figure 4:**
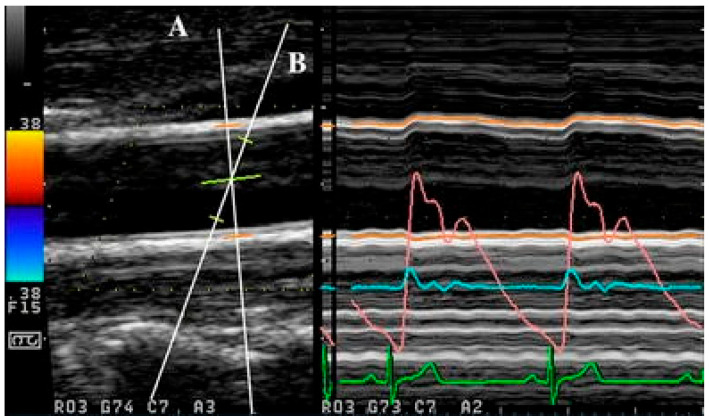
Simultaneous acquisition with two ultrasound beams (A and B), with overlap at the range gate region (from [156] with permission).

Apart from the use of singular ultrasound beams, as with the abovementioned methods, simultaneous acquisition can also be achieved with the use of multiple ultrasound beams, as demonstrated in [155,156]. In multiple beam acquisition, colour Doppler is used to obtain the blood flow velocity while diameter changes were measured using M-mode. The system consists of both the colour Doppler system and an echo-tracking subsystem that can use different ultrasound beams for velocity and diameter change, respectively. These beams can be independently manipulated, with an intersection between the beams at the range gate for both diameter and velocity measurement, as shown in Figure 4 [156]. From [155], this method was found to have low variabilities in the maximum velocity and arterial diameter measurements despite the need to manipulate different beams, which thereby supports its reproducibility.

In sum, simultaneous methods can circumvent the issue of alignment errors, albeit at the cost of greater computational demand.

### 6.3. Comparison and Evaluation of Methods

Table 2 provides a comparison of non-simultaneous and simultaneous methods in PWV parameter acquisition. In the acquisition of parameters for PWV measurement, non-simultaneous acquisition methods present as simpler and less computationally demanding options. However, a key limitation of non-simultaneous acquisition is the fact that velocity and diameter waveforms are not obtained at the same time [19,31]. This lack of simultaneity is a source of error in PWV assessment due to inaccurate time alignments between the two curves despite there being methods to ensure as close an alignment as possible. Additionally, the possibility of inter-cycle variations between diameter and velocity acquisition implies that, even with accurate time alignment [71], the measured diameter at each instance may not correspond to the velocity measured at the same timepoints. This issue may be overcome by estimating the velocity and diameter from the same data or with the use of one transducer angled oblique to the vessel and another perpendicular to the vessel for respective velocity and diameter measurement at the same region of interest [41,156].

In general, while simultaneous acquisition was more computationally complex, the advantage and limitations of each method varied. Of the discussed methods, the cross-correlation-based methods have a unique advantage in their ability to perform measurements with the use of B-mode imaging, which means that the transducer is kept in one position throughout acquisition. This ensures that the imaged region is constant, thereby preventing errors that may arise from Doppler angle adjustment. However, this method is computationally intense and may not be used in regions of excessively high velocities due to the limitations of the ultrasound system, which can be problematic for clinical use [5]. In contrast, while the dual ultrasound beam method required positioning of two beams, the results had good reproducibility [155,156]. Additionally, the ability to independently manipulate the beams may allow for flexibility in conducting measurement.

In all, while no one method is currently optimal, simultaneous acquisition methods have a distinct advantage in preventing errors due to delays or possible physiological differences between cardiac cycles. Each discussed method has advantages that can be leveraged for improving acquisition; hence, apart from comparisons between the methods, for future work, it is crucial to note the various means by which acquisition can be further improved. For instance, the use of ECG gating allows for consistent measurements over each cardiac cycle, which improves repeatability of the measurement. Meanwhile, the use of filters such as singular value decomposition to separate tissue signals can improve the signal–noise ratio in data acquisition, particularly in Doppler ultrasound, wherein noise can result in aliasing and affect velocity measurement.

## 7. Conclusions

With a focus on loop-based PWV estimation methods, this review sought to evaluate different approaches for ultrasound-based acquisition while considering technical parameters and physiological realities to optimise the accuracy, reliability, and reproducibility of the parameters collected for estimation. As such, this paper has summarised the use of ultrasound imaging in vessel data acquisition, covered the different ultrasound-based imaging modalities for acquiring area and velocity data and their developments, and compared simultaneous and non-simultaneous data acquisition for PWV estimation.

Based on the reviewed literature, despite its challenges and considerations, ultrasound remains a promising means by which PWV can be obtained non-invasively. It is key, therefore, to carefully adjust the key parameters when using the various imaging modalities for measurement, particularly in the use of Doppler ultrasound for velocity measurement. In the comparison of simultaneous and non-simultaneous acquisition, it is evident that there is no optimal method for data acquisition. However, simultaneous acquisition, once optimised, would allow for the acquisition of both diameter and velocity waveforms that prevents errors from arising due to physiological differences between two cardiac cycles.

Future developments in the use of ultrasound for PWV measurement will likely focus on improvements to be made in the acquisition phase as well as improvements in processing the ultrasound data post-acquisition. Firstly, experimental investigations between non-simultaneous and simultaneous methods can be conducted to better compare the accuracy and feasibility of each method and to improve upon the best-performing method. For other improvements in haemodynamic variable acquisition via ultrasound, the discussed challenges present possible avenues of work. Given that wave reflections can greatly affect data acquisition, further research into wave analysis methods to effectively separate forward and backward flow is warranted. Meanwhile, investigations into machine learning may mitigate errors arising from operator reliance by automating part of the acquisition process or providing feedback to operators during acquisition. Apart from addressing challenges, working around the limitations of ultrasound imaging is another area of improvement, and could include considering the use of media, developments in the design and piezoelectric crystal composition of the ultrasound transducer, or developing a standardised method to limit the effect of motion or differences in acoustic impedance and ensure consistently high data quality. Another potential area for exploration is improvement in processing of ultrasound data after acquisition, which could include determining the most accurate models for PWV estimation and developments in subsequent applications of PWV like pressure estimation.

Overall, ultrasound is a promising non-invasive alternative to the current non-invasive gold standard of carotid-femoral PWV (cfPWV) measurement via direct tonometry and further research needs to be conducted to overcome current constraints and ensure accuracy, reliability, and repeatability for clinical use.

## Figures and Tables

**Table 2:** Comparison of non-simultaneous and simultaneous acquisition methods for diameter and velocity.

Acquisition Type	Imaging Method and Key Acquisition Details	Advantages	Limitations	Reference
Non-simultaneous	B-mode imaging and PW Doppler imaging method. ECG gating used for time alignment.	The low computation demand and low complexity Application at different arterial sites is convenient	Requires a method to ensure proper alignment of haemodynamic loops. Delays, heart rate variations between measurement time periods of velocity and diameter or calculations with different timescales cause significant error and reduces precision	[31]
	M-mode imaging and PW Doppler imaging method. Ultrasound data is recorded over several cardiac cycles	Diameter distension measured more accurately and precisely with the use of M-mode		[19]
Simultaneous	M-mode imaging and PW Doppler imaging	Fairly reproducible results	Manual adjustment of gates and transducer angles are needed which may introduce errors in measurement.	[33,104,153]
	B-mode imaging with cross-correlation techniques	Use of B-mode imaging for both variables circumvents issues arising from manual transducer angling.	Unable to use if axial velocities are excessively high due to the limited framerate of the ultrasound system. More computationally demanding.	[5,34]
	M-mode imaging and colour Doppler with two ultrasound beams positioned at different angles	Independent manipulation of ultrasound beams for acquisition is possible with good reproducibility	Arterial movement due to normal artefacts such as respiration and pulsation may affect diameter measurements	[155,156]

## Data Availability

No new data were created or analyzed in this study. Data sharing is not applicable to this article.

## Abbreviation

ABP	Arterial Blood Pressure
CADMAS	Cross-Angular Delay Multiply and Sum
CCA	Common Carotid Artery
cfPWV	Carotid-Femoral Pulse Wave Velocity
CNN	Convolution Neural Network
CT	Computed Tomography
DMAS	Delay Multiply and Sum
ECG	Electrocardiography
FMAS	Filter Multiply and Sum
lnDU	lnDiameter-Velocity
MRI	Magnetic Resonance Imaging
PIV	Particle Image Velocimetry
PRF	Pulse Repetition Frequency
PUV	Perpendicular Ultrasound Velocimetry
PWI	Pulse Wave Imaging
PWV	Pulse Wave Velocity
QA	Flow-Area
RF	Radiofrequency
SVD	Singular Value Decomposition
TGC	Time Gain Compensation
WSA	Wave Separation Analysis
